# Study on the mechanism of erectile dysfunction in D-galactose induced aging rats: the construction of aging model

**DOI:** 10.1186/s40659-026-00705-x

**Published:** 2026-06-08

**Authors:** Taotao Sun, Yipiao Liu, Wenjia Deng, Yinwei Chen, Wenchao Xu, Penghui Yuan

**Affiliations:** 1 Department of Urology, The First Affiliated Hospital of Zhengzhou University, Zhengzhou, 450052 China; 2 Department of Plastic Surgery, The Third People’s Hospital of Zhengzhou, Zhengzhou, 450052 Henan China; 3 Reproductive Medicine Center, Tongji Hospital, Tongji Medical College, Huazhong University of Science and Technology, Wuhan, 430030 China; 4 Department of Urology, Traditional Chinese and Western Medicine Hospital of Wuhan,Tongji Medical College, Huazhong University of Science and Technology, Wuhan, 430030 China

**Keywords:** Erectile dysfunction, Aging, D-galactose, Oxidative stress, Cell cycle, Fibrosis

## Abstract

**Background:**

The incidence of erectile dysfunction (ED) rises sharply with age, which has brought a huge social burden. The exploration of aging-related ED is hindered due to the limitations of natural aging models. As an ideal inducer of accelerated aging models, the mechanism of D-galactose (D-gal) in ED has not been fully elucidated. Therefore, this study aimed to verify the feasibility of aging-related ED induced by D-gal and explore its potential mechanism.

**Methods:**

Primary corpus cavernosum smooth muscle cells (CCSMCs) and human umbilical vein endothelial cells (HUVECs) were incubated with concentration gradients of D-gal. Meanwhile, aging rat models were established via intraperitoneal and subcutaneous injection of D-gal, and the erectile function of all subjects was measured by electrical stimulation. Samples from both cells and animals were then collected for subsequent detection.

**Results:**

Cellular senescence was confirmed in both cells and was accompanied by oxidative stress, mitochondrial dysfunction, and cell cycle arrest in vitro. The erectile function of rats treated with D-gal was affirmed to be impaired compared with normal rats. In addition to the above-mentioned pathological alterations, tissue fibrosis was also associated with D-gal administration in vivo. Moreover, no significant difference was found between the two administration methods in inducing senescence of rats.

**Conclusions:**

D-gal could promote the progression of aging-related ED, accompanied by the exacerbation of oxidative stress, mitochondrial dysfunction, cell cycle arrest, and fibrosis. Our data suggested that D-gal may be an ideal mediator for the induced model of aging-related ED, which could facilitate preclinical exploration of this disease.

**Supplementary Information:**

The online version contains supplementary material available at 10.1186/s40659-026-00705-x.

## Introduction

Erectile dysfunction (ED) is defined as the failure to attain or sustain enough penile rigidity for satisfaction during coital activity. As a common male sexual dysfunction, ED affects the physical and mental health of both couples, especially among the elderly. Studies have shown that the incidence of ED in men over 40 years old is about 30%, and it gradually increases with age, even approaching 100% in people over 70 years old [1, 2]. In the context of the aging of the global population, ED would undoubtedly bring a greater social burden. In addition, given that the penis is the terminal part of the systemic vascular system, ED has been pointed out as an early indicator of cardiovascular disease, which is particularly important for the health management of the elderly population [3]. Therefore, it is imperative for the medical community to explore aging-related ED in depth.

The exploration of ED management strategies usually begins with animal studies, with rats often being the preferred research subjects in this process. The construction of aging models is generally divided into two categories: natural aging models and accelerated aging models [4]. Under natural aging conditions, it takes more than 20 months to construct an aging-related ED rat model [5]. As a result, the study of natural aging models is time-consuming, labor-intensive, costly, and has large individual differences in animals. Although the natural aging models are most consistent with the characteristics of human aging, the above drawbacks greatly limit our exploration of the pathogenesis of aging-related ED. In contrast, the induced accelerated models avoid the above shortcomings, making the acquisition of aging models more convenient, less time-consuming, and the aging effects are relatively more controllable [6]. For aging-related ED, D-galactose (D-gal) seems to be an ideal way to construct an induced acceleration model.

D-gal is a common small-molecule monosaccharide found naturally in the body and in daily foods [7]. However, when D-gal is at an excessively high level, it would be oxidized by galactose oxidase to produce reactive oxygen species (ROS), which accelerates the aging of the body. Another view is that high levels of D-gal are reduced to galactitol by aldose reductase, which cannot be further metabolized and accumulates in cells, affecting the normal cell osmotic pressure. This would lead to cellular dysfunction and cause the body to produce aging-like characteristics [8]. Compared with genetic engineering, total body irradiation, ozone and other aging induced methods, D-gal has unique advantages in timeliness, economy and safety. In addition, D-gal has been widely used in studies of the brain, heart, kidneys, liver, and testicles, which lays the foundation for D-gal induced aging-related ED [6, 9].

In this study, we employed concentration gradients of D-gal to intervene in corpus cavernosum smooth muscle cells (CCSMCs) and human umbilical vein endothelial cells (HUVECs), aiming to verify the effect of D-gal on cellular senescence. Additionally, animal models of aging in rats were constructed by intraperitoneal (IP) or subcutaneous (SC) injection of D-gal to further explore the characteristics of aging-related ED model induced by D-gal.

## Materials and methods

### Culture and group of cells

As described previously, we primary isolated CCSMCs from the corpus cavernosum of 8-week-old male Sprague-Dawley (SD) rats [10]. The cells were cultured in DMEM medium supplemented with 10% FBS and kept under normal conditions (5% CO_2_, 37℃). CCSMCs were separated from fibroblasts by different attachment rates and were identified by immunofluorescence (IF) for markers (α-SMA and desmin). As for HUVECs (ATCC, Manassas, VA, USA), cells were cultured with endothelial cell growth medium (ProCell, Wuhan, China) under the same conditions.

In the cell grouping, we first incubated CCSMCs and HUVECs in serum-free medium for 12 h. D-gal (Beyotime Biotechnology, Shanghai, China) at concentration gradients was used to induce cellular senescence and was incubated with cells for different time periods according to published literature [11, 12]. At the same time, the same concentration of mannitol (Beyotime Biotechnology) was employed to eliminate the influence of osmotic pressure. Cell Counting Kit (CCK-8; Yeasen, Shanghai, China) was then performed to evaluate the effects of D-gal and mannitol on cells under the above conditions referring to instructions. The appropriate concentration and duration of D-gal were finally determined and used in subsequent cellular experiments.

### Animals

The design of our experiment was approved by the Animal Welfare and Ethics Committee of Henan University of Chinese Medicine (NO. DWLL20220401579). A total of 24 eight-week-old male SD rats from Beijing Vital River Laboratory Animal Technology Co., Ltd. (Beijing, China) were included in this study. All rats were first acclimated for one week (without restriction of food and water). The sexual function of these rats was also verified to be normal through mating experiments before the intervention [10, 13].

The rats in this study were randomly divided into 3 groups (8 rats in each group) and received injection of D-gal for 8 weeks. One group was the DGAL-IP group (150 mg/kg/day, IP). Another group was the DGAL-SC group (150 mg/kg/day, SC). The remaining 8 rats were treated with saline as the control group. The dosage and frequency of D-gal administration were determined with reference to the relevant literature [14, 15].

### Evaluation of erectile function

After 3 days of drug washout, all rats were preliminarily judged for erectile function through the apomorphine (APO) experiment (100 µg/kg; SC) [16]. The number of erections in the rats within 30 min was recorded, which was positively correlated with erectile function. Subsequently, we used electrical stimulation of the cavernous nerve to measure the intracavernous pressure (ICP) of the animals. The arterial pressure was also recorded during the same period. These data could directly reflect differences in erectile function among rats [10, 13]. Then, the corpus cavernosum was cut into several sections. The morphologically intact part was paraffin-embedded, and the rest was frozen at -80 ℃. In addition, serum was also collected for subsequent testing.

### Senescence-associated β-galactosidase staining

Senescence-associated β-galactosidase (SA-β-gal) staining kit (Beyotime Biotechnology) was used to detect the activity of SA-β-gal in cells according to the manufacturer’s protocols. The dark blue product under the optical microscope represented SA-β-gal, and its proportion in all cells reflected the degree of cellular senescence.

### In vitro scratch assay

The migration ability of cells in vitro was measured by scratch assay. After the cells in the six-well plate were highly confluent, a 200 µl sterile pipette tip was used to leave a scratch in the culture well. Then, cell culture medium containing different concentrations of D-gal was replaced after washing with PBS solution. Next, the wound area was recorded 0, 24 and 48 h after the scratch. The cell migration ability under different concentrations of intervention was inferred by comparing the wound area.

### Flow cytometric analysis

Annexin V-FITC/PI apoptosis detection kit (Yeasen) was applied to detect the apoptosis level in each group of cells. The apoptosis rate was calculated to reflect the proportion of apoptotic cells. To analyze cell cycle, cell cycle analysis kit (Yeasen) was employed after cell harvesting. The distribution of cell cycle was statistically analyzed to evaluate the cell status.

### Western blot analysis

Proteins from CCSMCs and HUVECs were prepared and quantified. Details of the primary antibodies used in this study are listed in Table S1. The membrane was incubated with secondary antibodies of the corresponding species for 1 h. The target bands detected by the imaging system were finally quantified using Image J software.

### Quantification of mitochondrial DNA (mtDNA) copy number

Total DNA in CCSMCs and HUVECs was isolated using DNA extraction kit (Yeasen). Real-time quantitative PCR (Yeasen) was then performed to quantify the mtDNA copy number. The above data were analyzed with the method of 2^−ΔΔCT^ [17]. The primers for the mitochondrial gene ND1 (mtND1) and nuclear-encoded gene GAPDH are listed in Table 1.

**Table 1:** Details of the primers used in Real-time quantitative PCR

Genes	Primer sequences (5′→3′)
mtND1-Rat	F GCAGGACCATTCGCCCTATT
	R TCGAAAACGGGGGTAGGATG
mtND1-Huamn	F AACCCGCCACATCTACCATC
	R AGGAGGCCTAGGTTGAGGTT
GAPDH-Rat	F CCTACCCAAAGGGACATCTTCA
	R AACGACCCCTTCATTGACCTC
GAPDH-Human	F GAAAAAGGGCCCTGACAACTCT
	R TGGTTGAGCACAGGGTACTTT

### Detection of oxidative activity and mitochondrial dysfunction

The levels of malondialdehyde (MDA) and superoxide dismutase (SOD) in tissues and cells were detected with corresponding assay kits (Beyotime Biotechnology). The fluorescent probe of DCFH-DA (Yeasen) was co-incubated with CCSMCs and HUVECs to evaluate the level of ROS in cells. The fluorescent probe of DHE (Beyotime Biotechnology) was co-incubated with frozen sections of penile tissue to evaluate the level of ROS in tissues.

To identify mitochondrial dysfunction in cells, the fluorescent probe of MitoSOX (Yeasen), which specifically targets mitochondria, was used to determine superoxide levels within mitochondria. Furthermore, the fluorescent probe of JC-1 (Yeasen) was also co-incubated with CCSMCs and HUVECs. The ratio of red/green fluorescence intensity was used to measure mitochondrial membrane potential.

### Immunofluorescence

After fixation with 4% paraformaldehyde, the cell slides were incubated with the corresponding primary antibodies. Details of the antibodies are provided in Table S1. Then, the secondary antibodies and DAPI were used for counterstaining of the cell slides. The final result was obtained under a fluorescence microscope.

### Detection of nitric oxide (NO), cyclic guanosine monophosphate (cGMP) and Ca^2+^

Lysates from tissues and cells were prepared according to the respective instructions. Total levels of NO in penile tissue and HUVECs were detected using a total NO assay kit (Beyotime Biotechnology). The levels of cGMP in penile tissue and CCSMCs were determined with an ELISA kit (Mbbiology, Jiangsu, China). A calcium assay kit (Beyotime Biotechnology) was used to measure the concentration of Ca^2+^ in CCSMCs.

### Histologic staining

The preparation of tissue paraffin sections was a prerequisite for this part of the experiment. Immunohistochemistry (IHC) could reflect the expression differences of target molecules between different samples. H&E staining was applied to depict the overall appearance of the corpus cavernosum morphology. Masson trichrome staining also had the ability to reveal the morphology of the corpus cavernosum. The red area represented the smooth muscle, while the blue area represented the collagen. In addition, resorcinol-fuchsin staining was implemented to assess the level of elastin in penile tissue. The purple-black part represented elastic fibers, the red part represented collagen fibers, and the yellow part represented other components of the background.

### Transmission electron microscopy

Fresh tissue of corpus cavernosum was cut into blocks of approximately 1 mm^3^, which were then fixed, embedded, polymerized, sliced, and stained. The final acquisition images were achieved using a transmission electron microscope.

### Statistical analysis

The results in this study were expressed in the form of mean ± standard deviation. Data analysis was performed using GraphPad Prism (version 8.0), and *p* < 0.05 was considered statistically significant. The comparison between two samples was completed with unpaired t-test, while the comparison between multiple samples was completed with one-way anova analysis followed by Tukey’s post hoc test.

## Results

### D-gal induced the senescence of CCSMCs and HUVECs

**Fig. 1:**
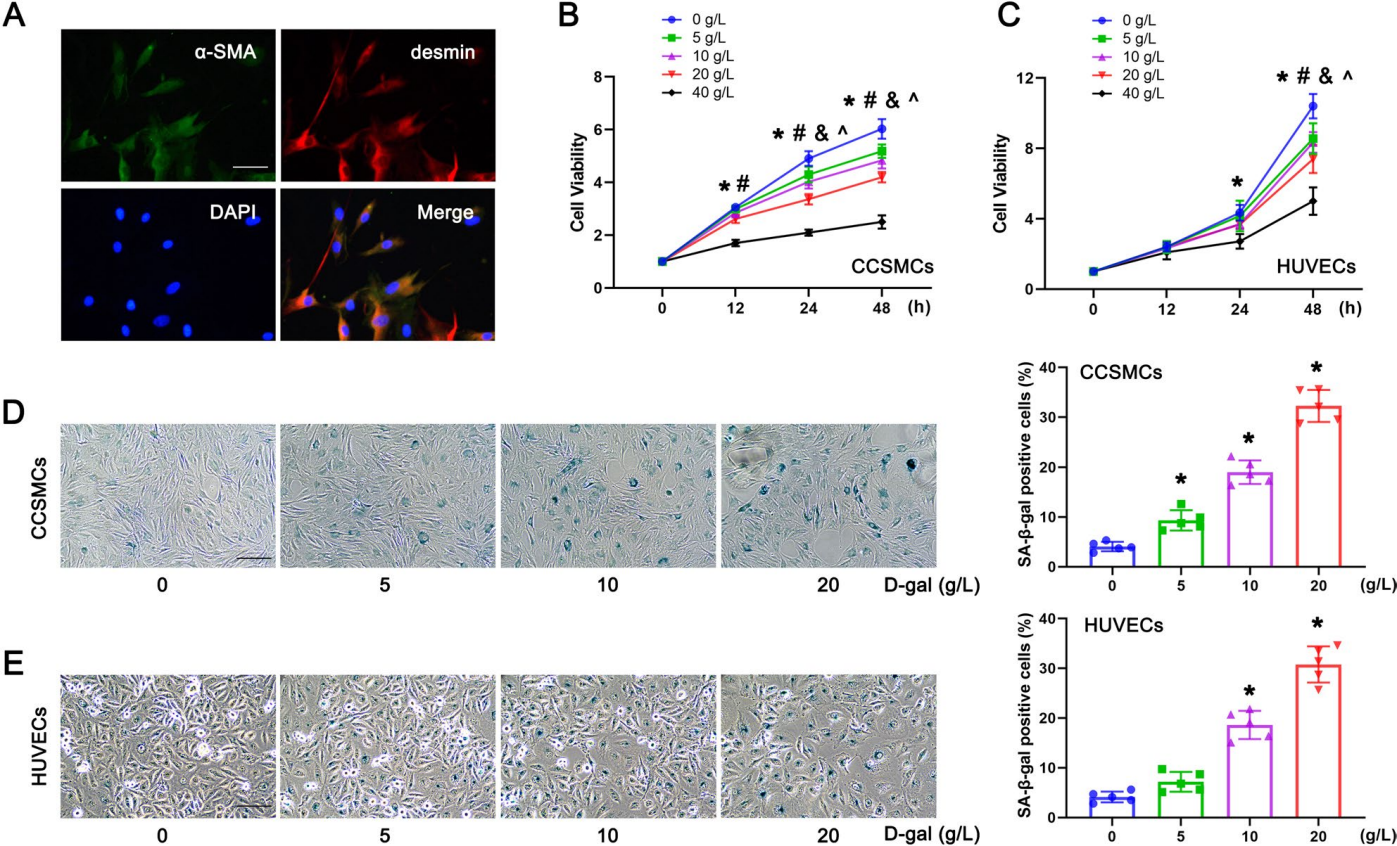
Effects of D-gal on inducing the senescence of CCSMCs and HUVECs. (**A**) Representative immunofluorescence of α-SMA and desmin in CCSMCs (× 400, bars = 50 μm). Cell viability of CCSMCs (**B**) and HUVECs (**C**) under concentration gradients of D-gal. *P < 0.05 of the 40 g/L group vs. the 0 g/L group; #*P* < 0.05 of the 20 g/L group vs. the 0 g/L group; &*P* < 0.05 of the 10 g/L group vs. the 0 g/L group; ^*P* < 0.05 of the 5 g/L group vs. the 0 g/L group. Representative images of SA-β-gal staining and the proportion of positive cells in different groups (**D**, × 100, bars = 200 μm; **E**, × 200, bars = 100 μm). *P < 0.05 vs. the 0 g/L group. D-gal, D-galactose; CCSMCs, corpus cavernosum smooth muscle cells; HUVECs, human umbilical vein endothelial cells

Normally functioning endothelial cells and smooth muscle cells are crucial in physiological erection of the penis [18]. The results of IF confirmed that the target cells were double positive for α-SMA and desmin, indicating the reliability of primary CCSMCs (Fig. 1A). According to the results of CCK-8, we found that the cell viability of CCSMCs and HUVECs decreased when the concentration of D-gal ranged from 0 to 40 g/L, and the above trend intensified over time (*P* < 0.05; Fig. 1B, C). However, the experiment of mannitol intervention suggested that the cells began to be affected by osmotic pressure when the concentration was equal to or greater than 40 g/L (*P* < 0.05; Figure S1A, B). Therefore, the concentration of D-gal in subsequent cellular experiments in this study was 0–20 g/L, and the incubation time was 48 h.

Under the influence of D-gal, enlarged volume and abnormal morphology were found in CCSMCs and HUVECs (Figure S1C, D). As one of the most easily recognizable and common signs of cellular senescence, the positive rate of SA-β-gal was increased when cells were co-incubated with D-gal (*P* < 0.05; Fig. 1D, E) [19]. In addition, the inhibitory effect of D-gal on the migration ability of cells had also been confirmed (*P* < 0.05; Figure S2). The above trend intensified with increasing concentration of D-gal.

### D-gal affected the cell cycle rather than apoptosis in vitro

**Fig. 2:**
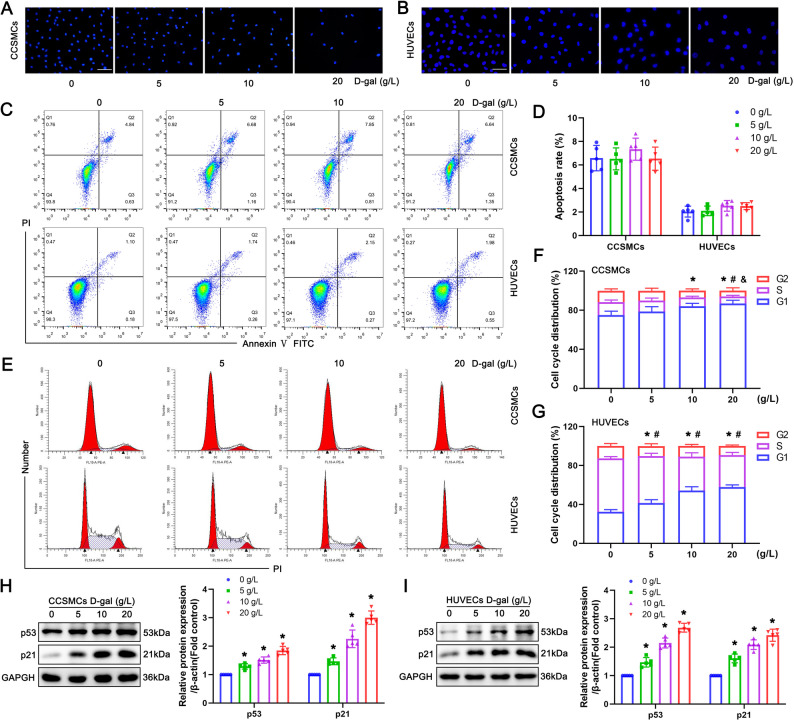
Effects of D-gal on affecting the cell cycle rather than apoptosis in vitro. Representative images of DAPI in CCSMCs and HUVECs for each group (**A**, × 200, bars = 100 μm; **B**, × 400, bars = 50 μm). Representative images of flow cytometry for apoptosis (**C**) and apoptosis rate (**D**) in different groups. Representative images of flow cytometry for cell cycle (**E**) and cell cycle distribution (**F**, **G**) in different groups. *P < 0.05 vs. the 0 g/L group in G1 phase; #*P* < 0.05 vs. the 0 g/L group in S phase; &*P* < 0.05 vs. the 0 g/L group in G2 phase. (**H**, **I**) Representative immunoblot and semi-quantification of p53 and p21 in different groups. *P < 0.05 vs. the 0 g/L group. D-gal, D-galactose; CCSMCs, corpus cavernosum smooth muscle cells; HUVECs, human umbilical vein endothelial cells

As shown in Fig. 2A, B, characteristics of apoptosis such as chromatin aggregation and nuclear fragmentation were not observed in cells exposed to different concentrations of D-gal. The results of flow cytometry for apoptosis also confirmed the above findings, that is, D-gal did not aggravate apoptosis. (Fig. 2C, D). Focusing on the cell cycle, we found that D-gal caused the arrest of the cell cycle in CCSMCs and HUVECs. The proportion of cells in G1 phase increased significantly after co-incubation with D-gal (*P* < 0.05; Fig. 2E–G). The results of western blot further confirmed that the protein levels of p53 and p21 were upregulated with increasing concentrations of D-gal (*P* < 0.05; Fig. 2H, I). The above evidence all indicated that D-gal would result in cell cycle arrest, but had no significant effect on apoptosis in CCSMCs and HUVECs.

### D-gal aggravated oxidative stress and mitochondrial dysfunction in cells

**Fig. 3:**
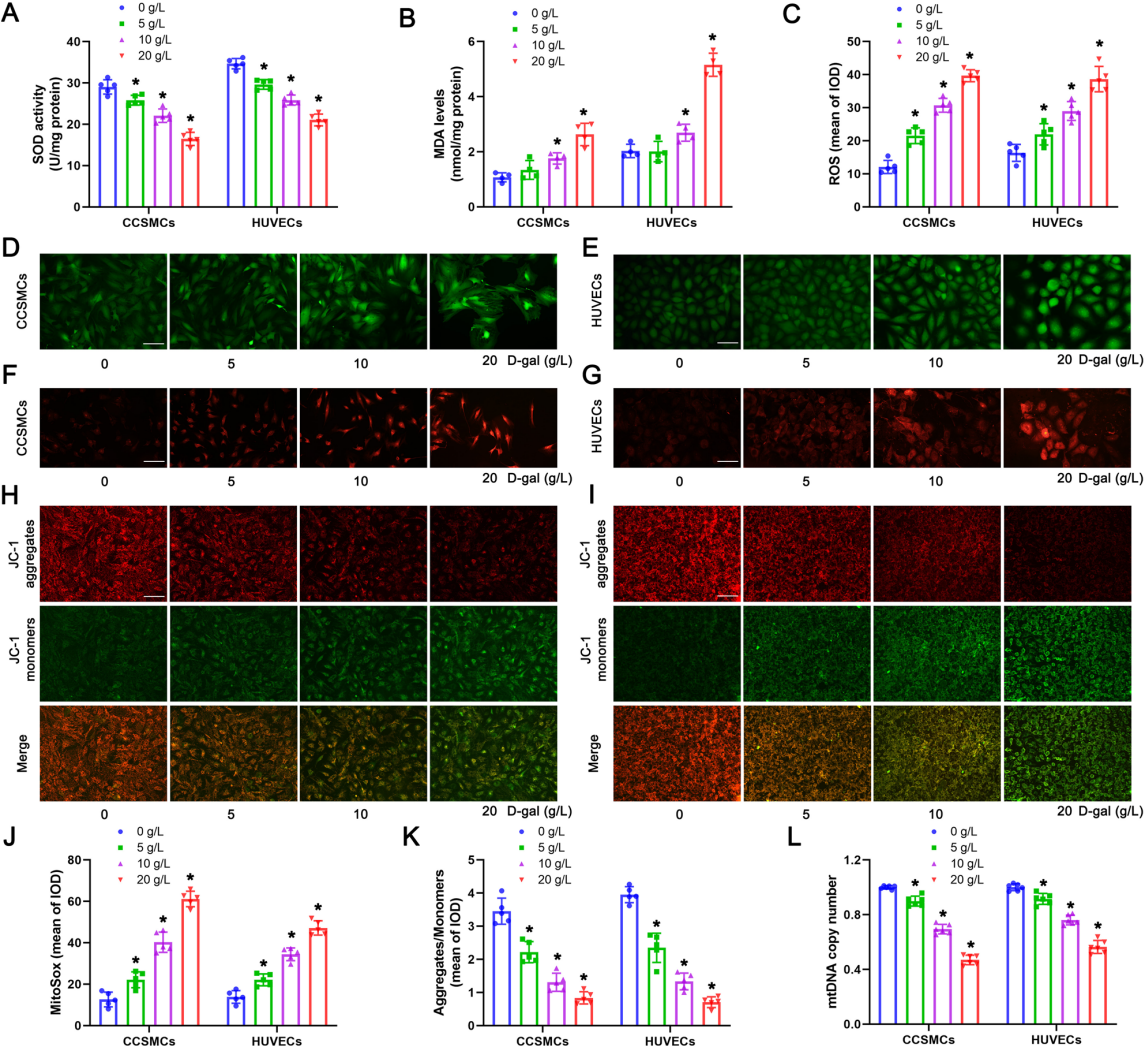
Effects of D-gal on aggravating oxidative stress and mitochondrial dysfunction in cells. Levels of SOD (**A**) and MDA (**B**) in CCSMCs and HUVECs for each group. Representative fluorescence (**D**, **E**) and semi-quantification (**C**) of ROS in different groups. Representative fluorescence (**F**, **G**) and semi-quantification (J) of Mitosox in different groups. Representative fluorescence (H, I) and semi-quantification (**K**) of JC-1 probe in different groups. (**L**) Levels of mtDNA copy number in different groups. **D**, **F**, **H**, **I**: × 200, bars = 100 μm; E, G: × 400, bars = 50 μm. *P < 0.05 vs. the 0 g/L group. D-gal, D-galactose; SOD, superoxide dismutase; MDA, malondialdehyde; ROS, reactive oxygen species; CCSMCs, corpus cavernosum smooth muscle cells; HUVECs, human umbilical vein endothelial cells; mtDNA, mitochondrial DNA

Intracellular oxidative and antioxidant substances jointly maintain the dynamic balance of oxidative activities. In Fig. 3A, the level of SOD decreased in the D-gal treatment groups, suggesting a decrease in antioxidant capacity in CCSMCs and HUVECs. On the contrary, the levels of MDA and ROS increased significantly after D-gal intervention, showing the upregulation of oxidative stress. (all *P* < 0.05; Fig. 3B-E).

As we know, mitochondria are the main site for the production of ROS in cells and play an important role in oxidative stress. The results of Mitosox fluorescence confirmed the accumulation of oxidative substances in mitochondria (*P* < 0.05; Fig. 3F, G, J). Abnormalities in mitochondrial membrane potential and mtDNA copy number also revealed D-gal-induced mitochondrial dysfunction (all *P* < 0.05; Fig. 3H, I, K, L). It should be noted that the IF results of 8-OHDG indicated that oxidative stress induced by D-gal may cause deep DNA damage (Figure S3).

### D-gal exacerbated the dysfunction of CCSMCs and HUVECs

**Fig. 4:**
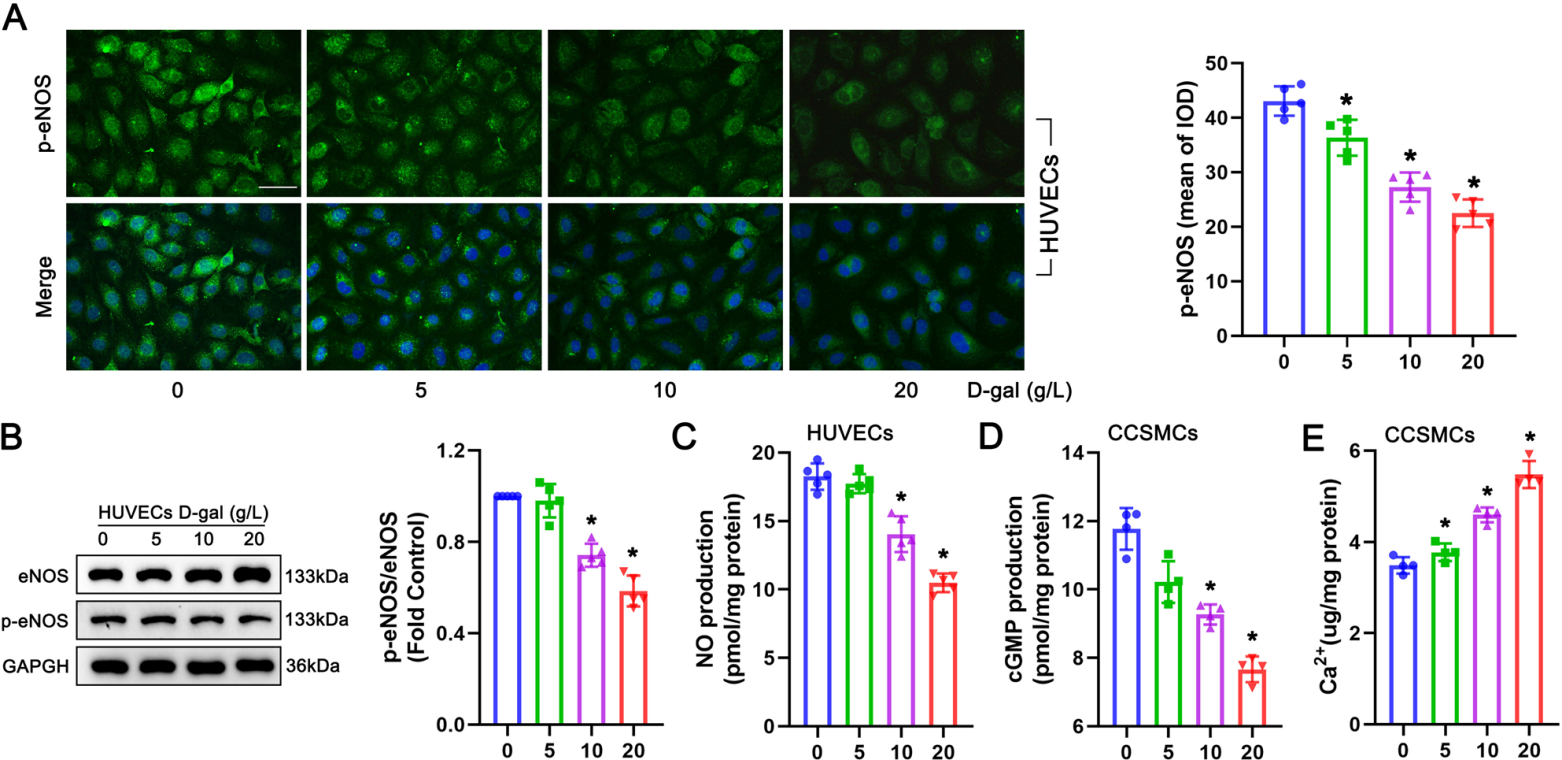
Effects of D-gal on exacerbating the dysfunction of CCSMCs and HUVECs. (**A**) Representative immunofluorescence and semi-quantification of p-eNOS in HUVECs for each group (× 400, bars = 50 μm). (**B**) Representative immunoblot and semi-quantification of eNOS and p-eNOS in HUVECs. The concentration of NO (**C**) in HUVECs and cGMP coupled with Ca^2+^ (**D**, **E**) in CCSMCs. *P < 0.05 vs. the 0 g/L group. D-gal, D-galactose; CCSMCs, corpus cavernosum smooth muscle cells; HUVECs, human umbilical vein endothelial cells; NO, nitric oxide; cGMP, cyclic guanosine monophosphate

To further verify the damage of D-gal on the cellular functions of CCSMCs and HUVECs, we treated HUVECs with concentration gradients of D-gal and subsequently cultured CCSMCs in the resulting conditioned medium [20]. The results of the experiment on HUVECs showed that the enzyme activity of eNOS reduced after D-gal treatment, accompanied by a decrease in NO production (all *P* < 0.05; Fig. 4A-C). The effect of D-gal on CCSMCs was manifested as a decrease in cGMP levels and an increase in Ca^2+^ concentrations (all *P* < 0.05; Fig. 4D, E).

### D-gal induced the senescence and erectile dysfunction in rats

**Fig. 5:**
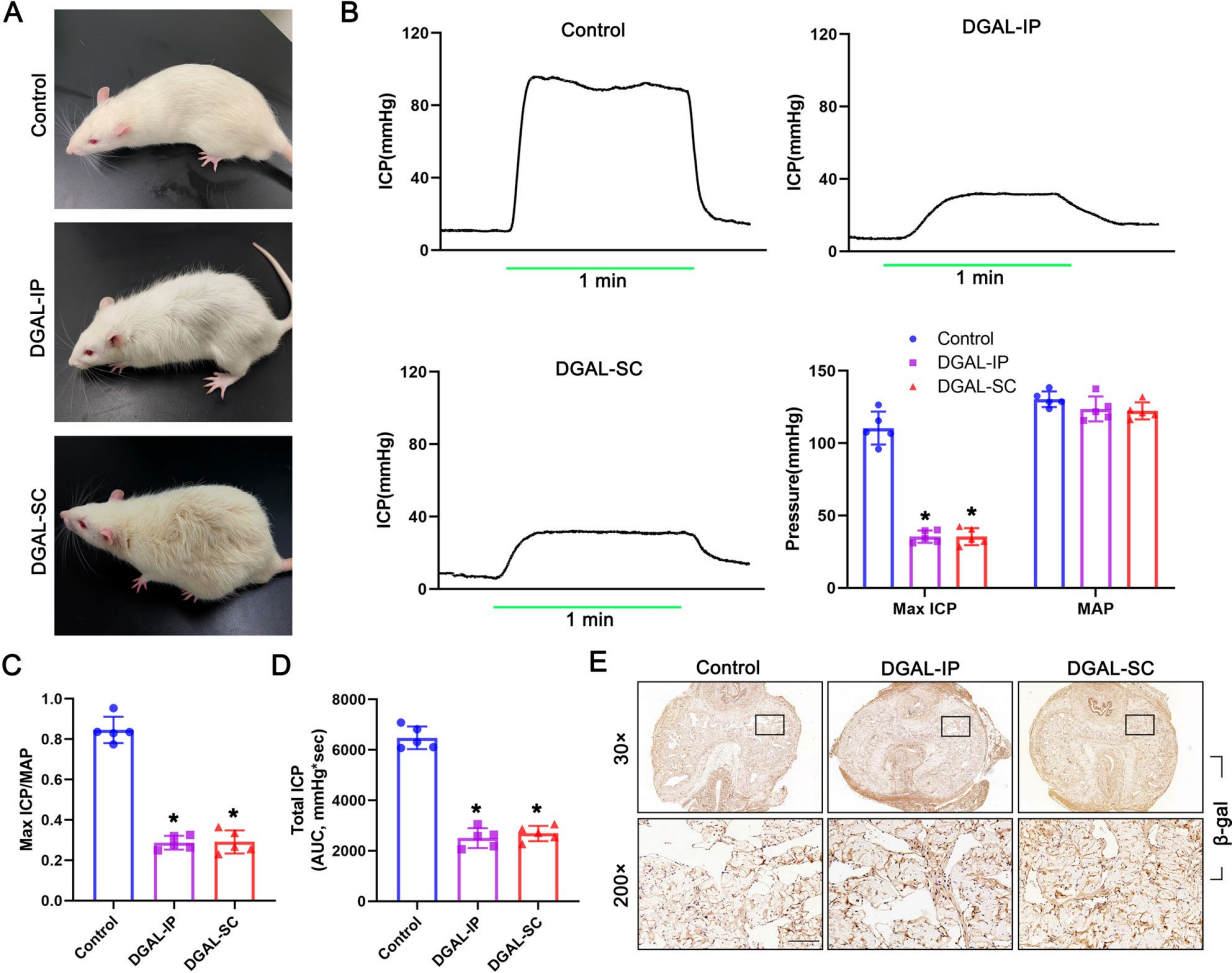
Effects of D-gal on inducing the senescence and erectile dysfunction in rats. (**A**) Representative images of physical appearance of rats in different groups. (**B**) Representative recordings and semi-quantification of ICP and arterial pressure for each group with electrical stimulation (15 Hz; 5.0 V; 1 min). The result of Max ICP/MAP (**C**) and total ICP (**D**) in different groups. (**E**) Representative immunohistochemistry of β-gal in different groups (bars = 100 μm). *P < 0.05 vs. the control group. D-gal, D-galactose; ICP, intracavernous pressure; MAP = mean arterial pressure; AUC = area under the curve; β-gal, β galactosidase

There were significant differences in the appearance of the rats between the groups. The hair of the rats in the control group was smooth and shiny, while that of the rats in the D-gal treated groups was rough and dull (Fig. 5A). In the APO experiment, the number of erections in the DGAL-IP and DGAL-SC groups was significantly lower than that in the normal group (Table 2). Further manometric data showed that the ratio of max ICP to mean arterial pressure (MAP) and total ICP of rats decreased after D-gal treatment (all *P* < 0.05; Fig. 5B–D). Besides, the results of IHC suggested that D-gal significantly increased the expression of galactosidase in the corpus cavernosum (Fig. 5E). In terms of metabolic and physiological parameters, there were no differences in body weight, fasting blood glucose, total cholesterol, triglycerides, high density lipoprotein, low density lipoprotein and testosterone between the groups (Table 2).

**Table 2:** Assessment of metabolic and physiological parameters of rats

	Control	DGAL-IP	DGAL-SC
Body weight (g), mean ± SD	571.83 ± 24.65	532.17 ± 30.62	579.67 ± 47.75
Fasting blood glucose (mmol/L), mean ± SD	5.22 ± 0.67	5.72 ± 0.69	5.77 ± 0.25
Number of erection, median (range)	3 (2–4)	0 (0–1)*	1 (0–1)*
Total cholesterol (mmol/L), mean ± SD	1.97 ± 0.17	1.69 ± 0.14	1.72 ± 0.15
Triglycerides (mmol/L), mean ± SD	0.76 ± 0.30	1.10 ± 0.78	1.07 ± 0.90
High density lipoprotein (mmol/L), mean ± SD	0.68 ± 0.08	0.52 ± 0.11	0.58 ± 0.03
Low density lipoprotein (mmol/L), mean ± SD	0.20 ± 0.05	0.20 ± 0.02	0.21 ± 0.05
Testosterone (pg/ml), mean ± SD	1480.84 ± 212.26	1431.70 ± 413.55	1434.02 ± 145.85

IP, intraperitoneal; SC, subcutaneous

*P < 0.05 vs. the control group

### D-gal intensified the level of fibrosis in the penis

**Fig. 6:**
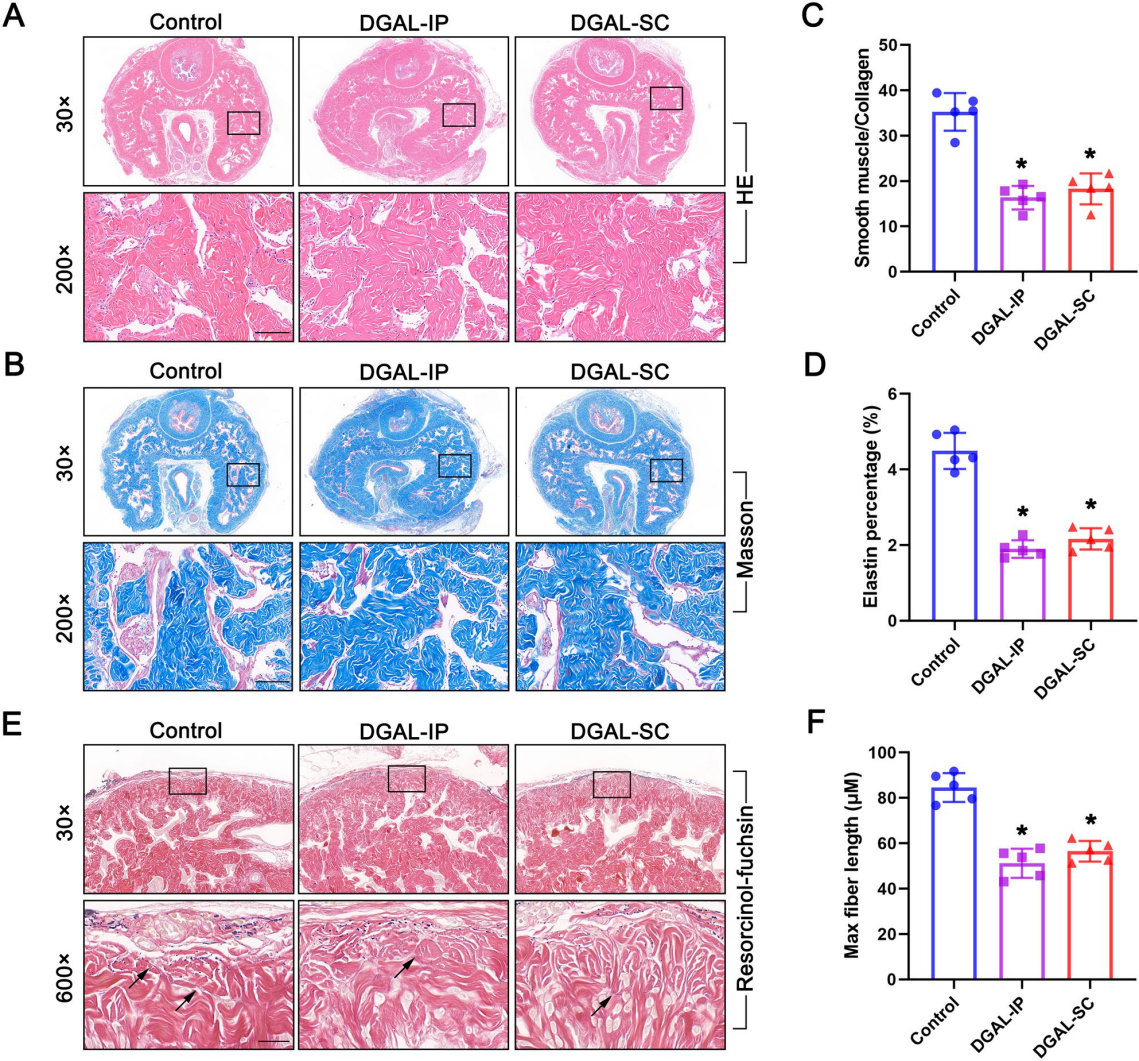
Effects of D-gal on intensifying the level of fibrosis in the penis. (**A**) Representative images of H&E staining in the penis for each group. Representative images (**B**) and semi-quantification (**C**) of masson trichrome staining in different groups. Representative images (**E**) and semi-quantification (**D**, **F**) of resorcinol-fuchsin staining in different groups. **A**, **B**: bars = 100 μm; E: bars = 30 μm. The black arrows indicate elastic fibers. *P < 0.05 vs. the control group. D-gal, D-galactose

Regardless of IP or SC, D-gal resulted in structural disorder of the corpus cavernosum, which is manifested by abnormal morphology of the blood sinusoids accompanied by thinning and fragmentation of the smooth muscle layer (Fig. 6A). The results of Masson trichrome staining showed that the ratio of smooth muscle to collagen was reduced in the DGAL-IP and DGAL-SC groups (*P* < 0.05; Fig. 6B, C). The detection of elastin also conformed to the above trend, that is, the content of elastin and the maximum elastic fiber length in the corpus cavernosum decreased after D-gal treatment (all *P* < 0.05; Fig. 6D–F).

### D-gal aggravated oxidative stress and mitochondrial dysfunction in rats

**Fig. 7:**
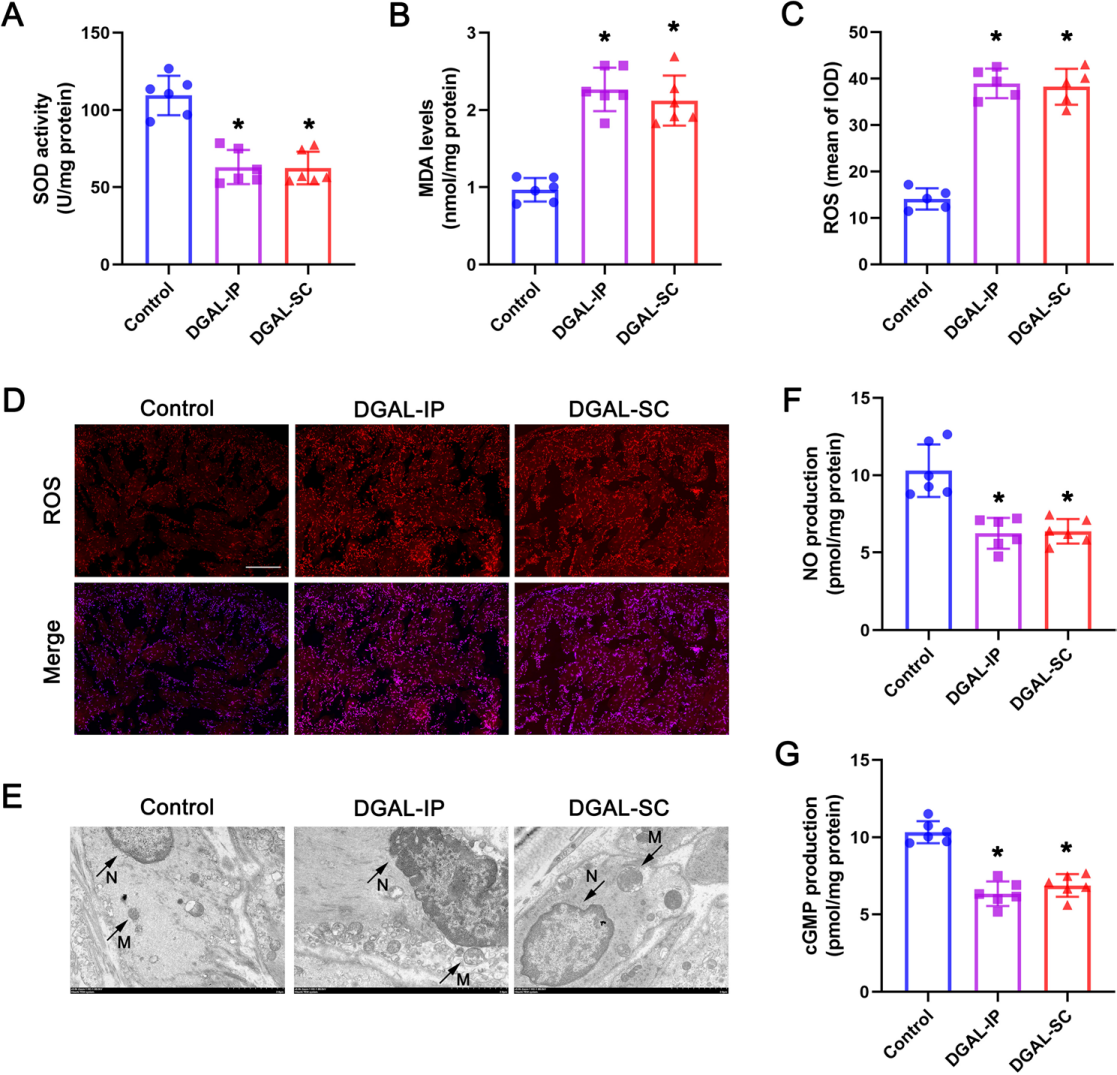
Effects of D-gal on aggravating oxidative stress and mitochondrial dysfunction in rats. Levels of SOD (**A**) and MDA (**B**) in the penis for each group. Representative fluorescence (**D**) and semi-quantification (**C**) of ROS in different groups. (**E**) Representative images of transmission electron microscopy in different groups. The black arrows indicate the nucleus “N” and mitochondria “M”. The concentration of NO (**F**) and cGMP (**G**) in different groups. D: × 100, bars = 200 μm. *P < 0.05 vs. the control group. D-gal, D-galactose; SOD, superoxide dismutase; MDA, malondialdehyde; ROS, reactive oxygen species; NO, nitric oxide; cGMP, cyclic guanosine monophosphate

After D-gal treatment, the level of SOD in the penis decreased, while the levels of MDA and ROS increased (all *P* < 0.05; Fig. 7A–D). Infolded nuclear membrane, swollen mitochondria and ruptured crista were found in transmission electron microscopy observations (Fig. 7E). Subsequently, the production of NO and cGMP was also affected, showing a significant decrease in the DGAL-IP and DGAL-SC groups (all *P* < 0.05; Fig. 7F, G).

## Discussion

Given the aging of the global population, the mechanisms and management strategies of aging-related ED are being gradually explored. The natural aging models have been criticized due to various defects. As an ideal inducer of aging models, whether D-gal could be used to construct the model of aging-related ED and its potential mechanism are still not sufficiently demonstrated. In order to provide more sufficient evidence for the above scientific issues, we explored the effect of D-gal on erectile function in vivo and in vitro in this study. The results showed that D-gal indeed induced ED. Further exploration revealed cell cycle arrest, oxidative stress and mitochondrial dysfunction under D-gal treatment. In addition, dysfunction of endothelial and smooth muscle cells and tissue fibrosis were also triggered.

At present, D-gal has been widely used in the construction of aging animal models, and certain achievements have certainly been made in this process. Studies have found that chronic administration of D-gal would induce brain aging, accompanied by neuronal damage and cognitive decline [21]. As the liver is the main organ for metabolism of D-gal, excessive intake of D-gal seriously affects liver function and lead to liver aging [22]. Hong et al. also pointed out that D-gal could mediate decreased cardiac function, shortened telomere length and cardiac aging [23]. As for the male reproductive system, injection of D-gal has been shown to accelerate testicular aging and lead to a decline in sperm quality [24]. Focusing on erectile function, several literatures have confirmed that D-gal induced aging models and exacerbates erectile dysfunction in rats [25, 26]. However, research on D-gal induced aging-related ED is still in its infancy. The above-mentioned existing evidence is limited to the outcome of D-gal causing aging, and lacks the exploration of detailed pathogenic mechanisms. This study once again confirmed that D-gal does induce aging-related ED in rats, and further conducted more detailed in vitro experiments to explore potential pathogenic factors. Furthermore, we used two common methods, IP and SC, to intervene in rats. The results found that the injection method did not seem to affect the severity of ED, which is consistent with the widespread adhibition of these two routes of administration in previous studies [9].

Excess D-gal in the body generates a large amount of ROS after metabolism and mediates oxidative stress, which seems to be the key mechanism of D-gal-induced aging. Multiple documents have confirmed the above view and found that inhibiting oxidative stress delayed the aging process induced by D-gal to a certain extent [9, 21, 22, 24, 27]. High concentration of D-gal in cells may change the osmotic pressure and cooperate with overloaded ROS to cause mitochondrial dysfunction. At the same time, damaged mitochondria in turn produce more ROS, which is mutually causal with oxidative stress [9, 28, 29]. Our in vitro and in vivo experimental data suggested the existence of D-gal-induced oxidative stress and mitochondrial dysfunction. Moreover, our results also preliminarily indicated the possibility of oxidative DNA damage, which is consistent with previous conclusions but requires more evidence to support [23, 27].

As a permanent state of cell cycle arrest, cellular senescence is triggered by various stresses. D-gal has been reported to stimulate cell cycle arrest and inhibit cell proliferation [30, 31]. Increased G1 phase and upregulation of p53 and p21 expression are common markers of cellular senescence [19]. Data from our in vitro experiments also showed that cell cycle arrest became more severe with increasing D-gal concentrations. Interestingly, the apoptosis rate in this study did not change before and after D-gal treatment, which is contrary to the results of previously published literature on D-gal-induced senescence of CCSMCs [26]. Of course, there are other literatures that are consistent with our results [28]. After careful comparison, we speculate that the above controversy is caused by the differences in the concentration and duration of D-gal intervention. Besides, oxidative stress is one of the critical factors that cause cell cycle arrest in cellular senescence. The inhibition of oxidative stress accelerates the cell cycle process and thus exerts an anti-aging effect [27].

Extracellular matrix changes and the resulting fibrosis are another recently proposed hallmark of aging [32]. In animal models of D-gal-induced aging, tissue fibrosis is also a common consequence [9]. The reduction of smooth muscle content and the accumulation of collagen are important characteristics of penile fibrosis. The destruction of the blood sinuses and elastic fibers of the corpus cavernosum is also not conducive to physiological erection [2, 13]. In our study, we did find the above changes, suggesting that D-gal could exacerbate the level of penile fibrosis. It is worth mentioning that oxidative stress is known to contribute to tissue fibrosis in the corpus cavernosum [33]. Inhibiting oxidative stress has also been shown to alleviate D-gal-mediated tissue fibrosis [34]. All of these evidences pointed to the importance of oxidative stress in D-gal-induced fibrosis.

**Fig. 8:**
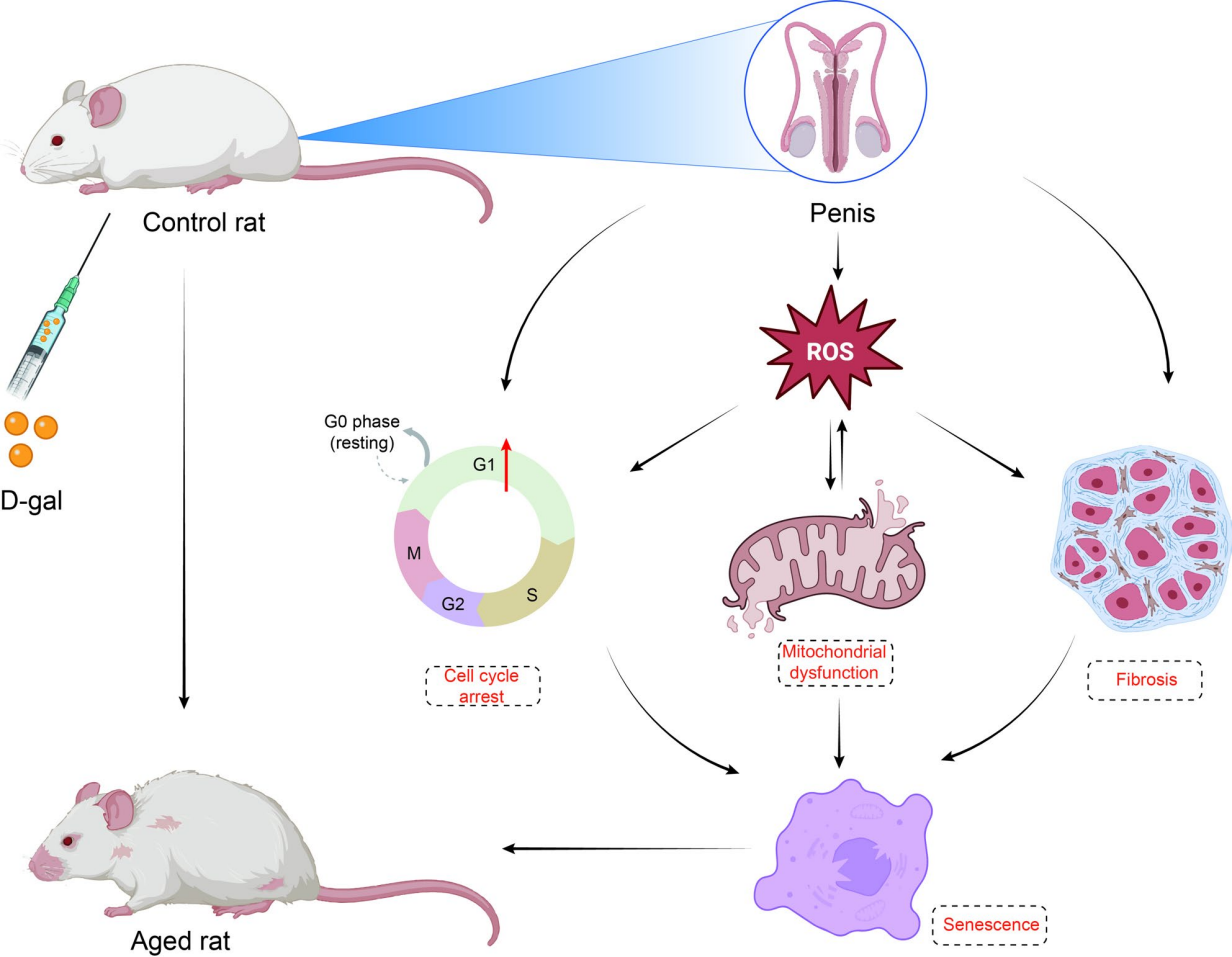
Potential mechanism of aging-related ED induced by D-gal. Rats exhibit impaired erectile function and aging appearance after D-gal supplementation. Upregulation of oxidative stress, mitochondrial dysfunction, cell cycle arrest and fibrosis are all involved in this process. Moreover, overloaded oxidative stress further aggravates pathological alterations in the penis. Created with BioRender.com. D-gal, D-galactose; ROS, reactive oxygen species

Based on the above data, we summarized the possible potential mechanisms by which D-gal contributed to aging-related ED (Fig. 8). Overloaded D-gal was metabolized to generate excessive ROS, which aggravated oxidative stress and mediated the occurrence of mitochondrial dysfunction. Cell cycle arrest and tissue fibrosis also occurred after D-gal intervention. At the same time, oxidative stress could further promote the process of cell cycle arrest and tissue fibrosis. The above alterations jointly accelerated the occurrence and development of aging-related ED after D-gal administration. It is worth noting that the accelerated aging model induced by D-gal has shown several hallmarks of natural aging, but the two models are still not entirely the same. Naturally aging animals could best simulate the human aging process. However, their extremely high cost, significant individual variability, and lengthy experimental cycles often limit their application in large-scale mechanistic studies. In contrast, the D-gal induced model is a relatively mature accelerated aging model that avoids the shortcomings of the aforementioned natural aging models [6]. Rats under D-gal administration exhibit aged appearance and impaired erectile function. The results of in vitro and in vivo experiments also conform to the characteristics of aging. The D-gal induced ED model provides an efficient and reliable platform for studying aging-related ED. Nevertheless, the effects of D-gal on factors such as nerves, blood vessels, and classical ED-related signal transduction pathways during penile erection are not yet fully elucidated and require further investigation.

Although we have demonstrated that D-gal contributed to senescence and impaired erectile function, some limitations still existed in this study. Firstly, we performed the method of conditioned medium to explore the interaction between CCSMCs and HUVECs. The effects of other co-culture methods such as transwell chambers may be more direct. Another limitation is the use of HUVECs as the in vitro endothelial model. Although HUVECs are a well-established model for conducting in vitro studies related to ED, they may not be able to fully reproduce the unique physiological characteristics of the cavernous endothelium. In addition, the animal experiments need further improvement, such as exploring the optimal duration and dose of D-gal administration, and the experimental verification of mitochondrial function in the corpus cavernosum. The above limitations are expected to be resolved in further exploration.

## Conclusions

D-gal was confirmed to induce the construction of aging-related ED model in vivo and in vitro. Further studies revealed the occurrence of oxidative stress and mitochondrial dysfunction under D-gal administration, accompanied by cell cycle arrest and fibrosis. Our study provided a theoretical basis for the construction of aging-related ED model induced by D-gal, and facilitated the exploration of the mechanism and management strategy of aging-related ED.

## Supplementary Information

Below is the link to the electronic supplementary material.

Supplementary Material 1.

## Data Availability

Data are available from the corresponding authors upon reasonable request.

## Abbreviations

ED: Erectile dysfunction
D-gal: D-galactose
CCSMCs: Corpus cavernosum smooth muscle cells
HUVECs: Human umbilical vein endothelial cells
ROS: Reactive oxygen species
IP: Intraperitoneal
SC: Subcutaneous
SD: Sprague-Dawley
IF: Immunofluorescence
APO: Apomorphine
ICP: Intracavernous pressure
SA-β-gal: Senescence-associated β-galactosidase
mtDNA: Mitochondrial DNA
mtND1: Mitochondrial gene ND1
MDA: Malondialdehyde
SOD: Superoxide dismutase
IHC: Immunohistochemistry
