# Supplementary material for: Study on the mechanism of erectile dysfunction in D-galactose induced aging rats: the construction of aging model
Source: Biol Res. 2026 Jun 8;59:45. doi: 10.1186/s40659-026-00705-x (PMC13471410; doi:10.1186/s40659-026-00705-x)
Supplement: Supplementary file 1 [file 40659_2026_705_MOESM1_ESM.pdf]

Supplementary Materials

Supplemental Table 1 The details of the antibodies used in the study

	Name	Abbreviation	concentration	source
WB	anti-p53	p53	1:1000	A16989; ABclonal, Wuhan, China
	anti-p21	p21	1:1000	A19094; ABclonal
	anti-eNOS	eNOS	1:1000	ab5589; Abcam, Cambridge, MA, USA
	anti-p-eNOS	p-eNOS	1:500	9571; Cell Signaling Technology (CST), Danvers, MA, USA
	anti-GAPDH	GAPDH	1:1000	GB11002; Servicebio, Wuhan, China
IF	anti-α-SMA	α-SMA	1:500	GB111364; Servicebio
	anti-desmin	desmin	1:1000	GB11081; Servicebio
	anti-p-eNOS	p-eNOS	1:200	AF3247; Affinity, USA
	anti-8-OHDG	8-OHDG	1:100	sc-66036; Santa Cruz Biotechnology, Dallas, Texas, USA
IHC	anti-β-gal	β-gal	1:100	15518-1-AP; Proteintech, Wuhan, China

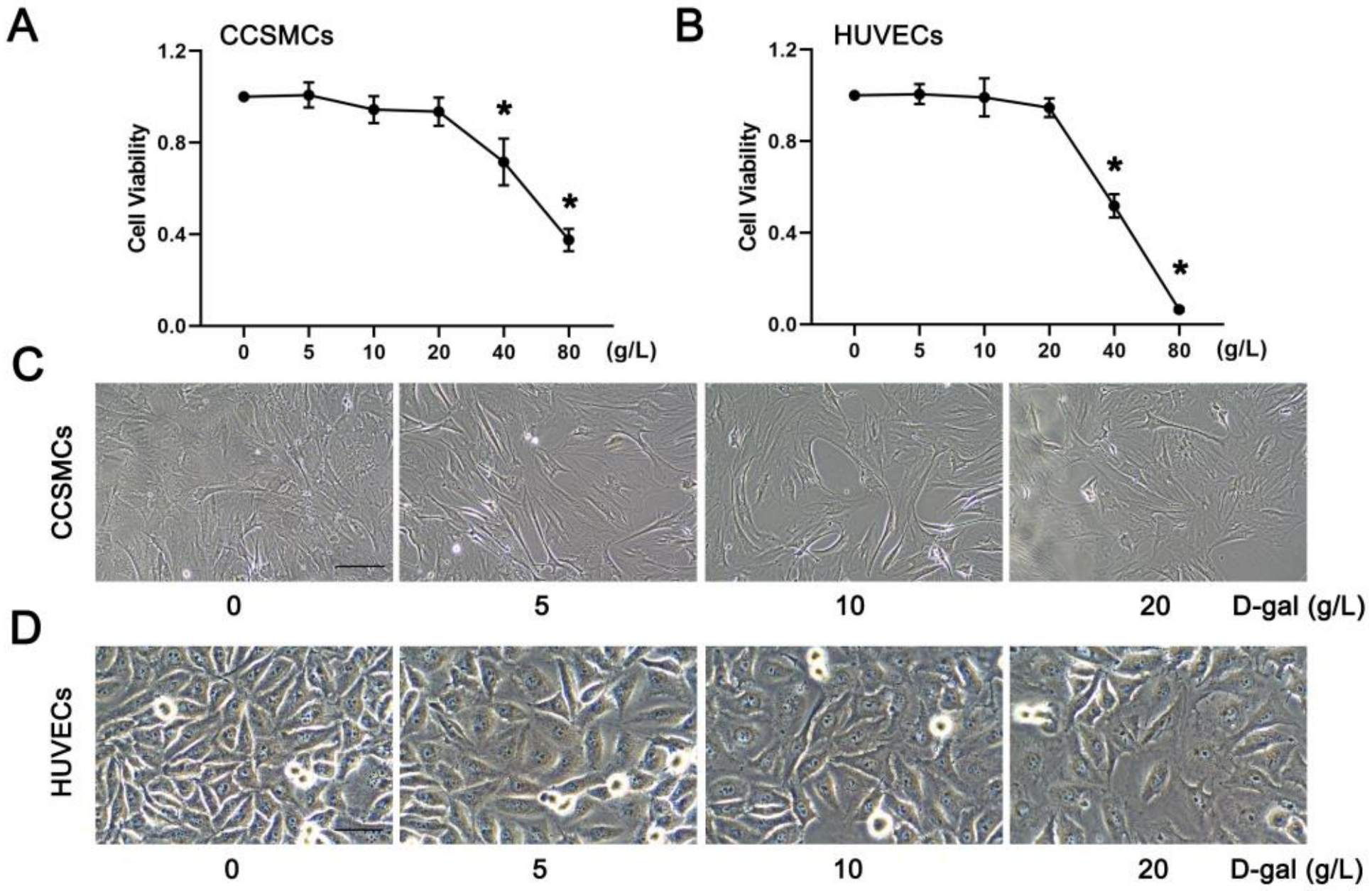

Supplemental Figure 1 Effects of mannitol on proliferation and D-gal on cellular morphology in cells. Cell viability of CCSMCs (A) and HUVECs (B) under concentration gradients of mannitol. Representative images (C, × 200, bars = 100 µm; D, × 400, bars = 50 µm) of cellular morphology in CCSMCs and HUVECs for each group. *P<0.05 vs. the 0 g/L group. D-gal, D-galactose; CCSMCs, corpus cavernosum smooth muscle cells; HUVECs, human umbilical vein endothelial cells.

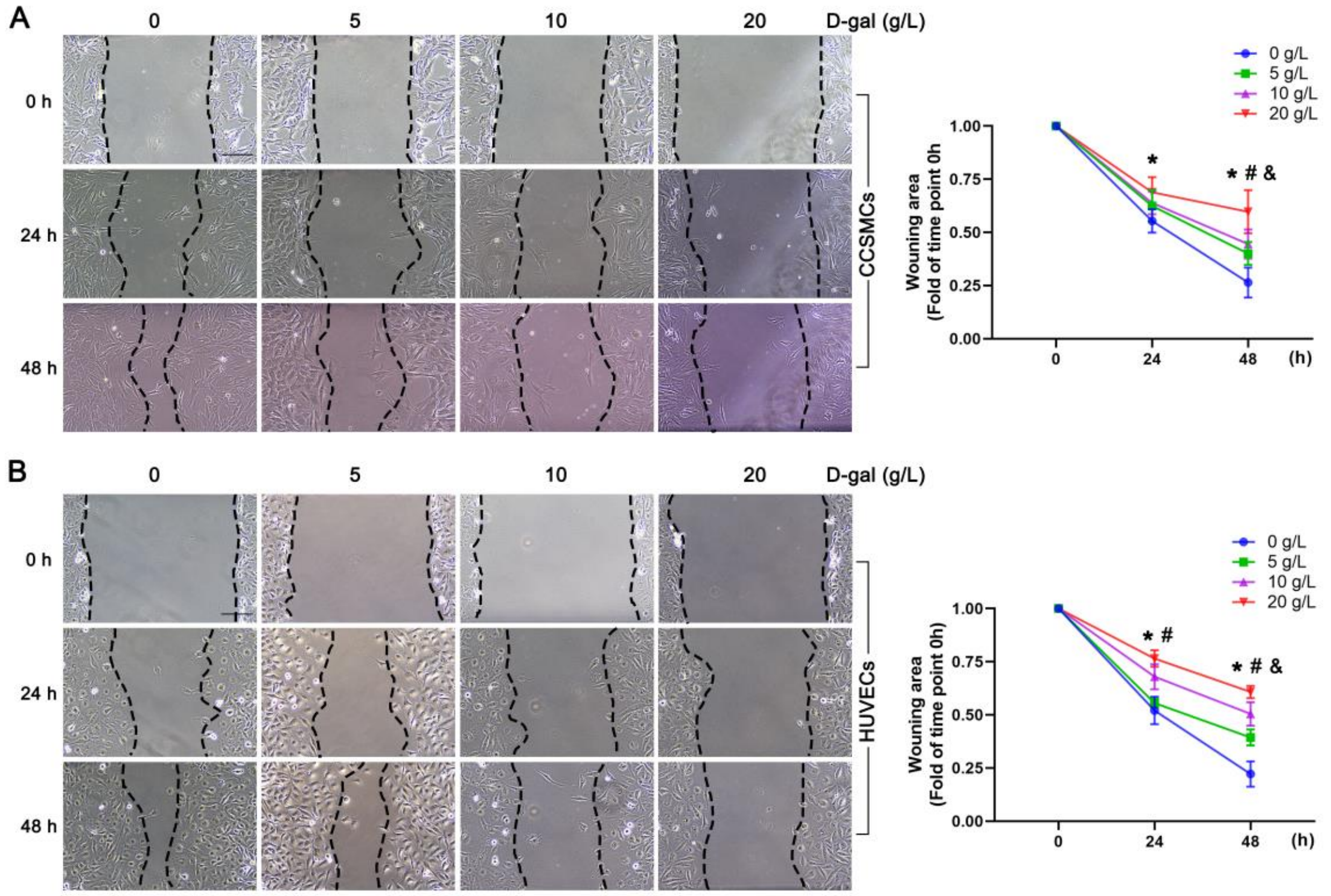

Supplemental Figure 2 Effects of D-gal on migration ability of CCSMCs and HUVECs. (A, B) Representative images (× 100, bars = 200 μm) of scratch assay for each group. *P<0.05 of the 20 g/L group vs. the 0 g/L group; #P<0.05 of the 10 g/L group vs. the 0 g/L group; &P<0.05 of the 5 g/L group vs. the 0 g/L group; D-gal, D-galactose; CCSMCs, corpus cavernosum smooth muscle cells; HUVECs, human umbilical vein endothelial cells.

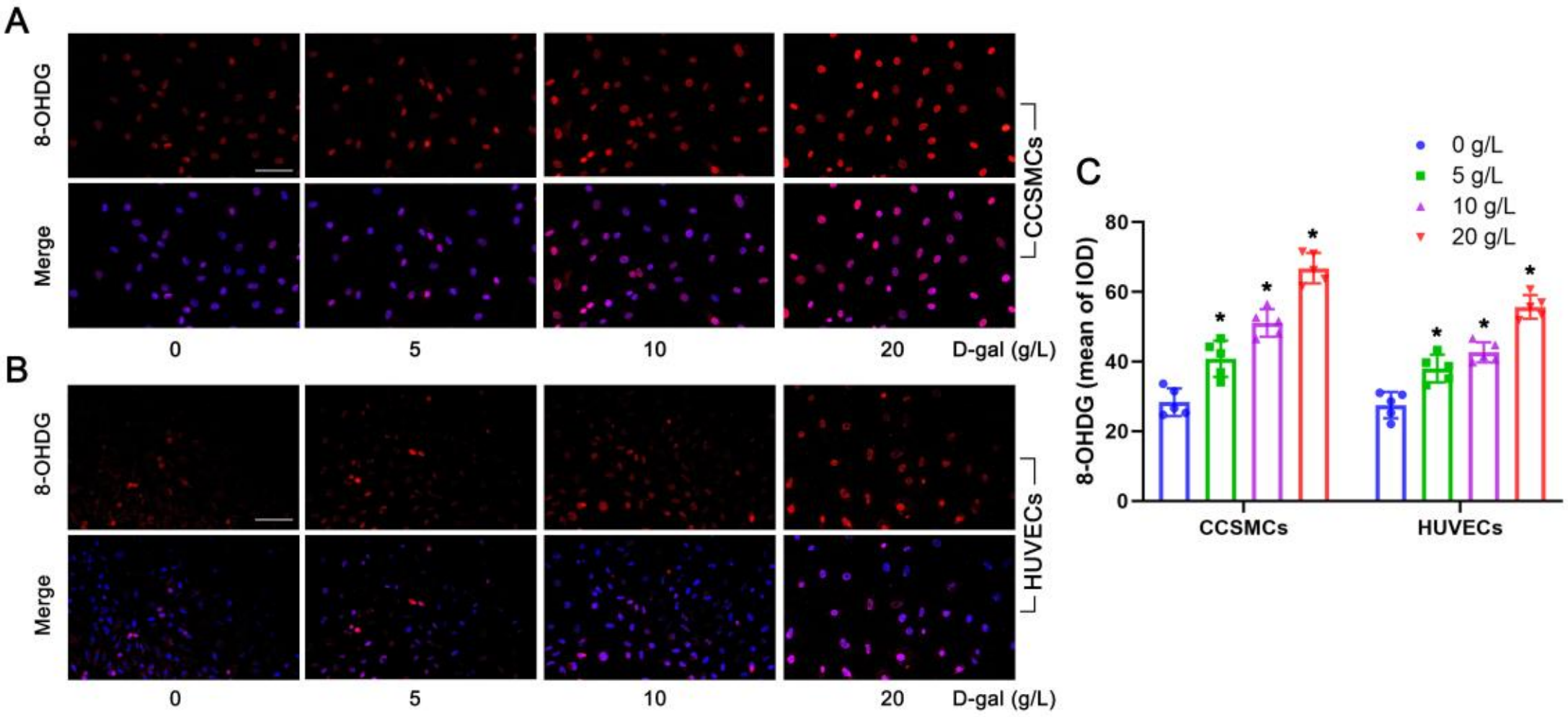

Supplemental Figure 3 Effects of D-gal on DNA damage of CCSMCs and HUVECs. Representative immunofluorescence (× 200, bars = 100 μm) (A, B) and semi-quantification (C) of 8-OHDG for each group. *P<0.05 vs. the 0 g/L group. D-gal, D-galactose; CCSMCs, corpus cavernosum smooth muscle cells; HUVECs, human umbilical vein endothelial cells.
